# Towards clinical magnetic particle imaging: safety measurements of medical implants in a human cadaver model

**DOI:** 10.1038/s44172-025-00561-9

**Published:** 2025-12-01

**Authors:** Franz Wegner, Thomas Friedrich, Patrick Naoki Elfers, Florian Kleefeldt, Dominik Peter, Philipp Gruschwitz, Teresa Reichl, Johanna Günther, Thomas Kampf, Martin A. Rückert, Volker C. Behr, Thorsten M. Buzug, Roman Kloeckner, Jörg Barkhausen, Thorsten A. Bley, Patrick Vogel, Viktor Hartung

**Affiliations:** 1 Institute of Interventional Radiology, University Hospital Schleswig-Holstein, Lübeck, Germany; 2 Fraunhofer IMTE, Fraunhofer Research Institution for Individualized and Cell-Based Medical Engineering IMTE, Lübeck, Germany; 3 Institute of Radiology and Nuclear Medicine, University Hospital Schleswig-Holstein, Lübeck, Germany; 4 Institute of Anatomy and Cell Biology, University of Würzburg, Würzburg, Germany; 5 Department of General, Visceral, Transplant, Vascular and Pediatric Surgery, Center of Operative Medicine, University Hospital Würzburg, Würzburg, Germany; 6 Department of Diagnostic and Interventional Radiology, University Hospital Würzburg, Würzburg, Germany; 7 Department of Experimental Physics 5 (Biophysics), Julius-Maximilians-University, Würzburg, Germany; 8 phase VISION GmbH, Rimpar, Germany; 9 Department of Diagnostic and Interventional Neuroradiology, University Hospital Würzburg, Würzburg, Germany; 10 Institute of Medical Engineering, University of Lübeck, Lübeck, Germany

**Keywords:** Tomography, Diagnostic devices

## Abstract

Magnetic Particle Imaging (MPI) is a preclinical imaging modality with potential for future clinical usage. The radiation-free guidance of endovascular interventions with MPI is especially promising. Here, we present a safety study on the heating of metallic medical implants during MPI measurements under realistic conditions in an extracorporeally-perfused cadaver model. The measurements were conducted by fiberoptic thermometers and showed no detectable heating of the tested endovascular devices in the cadaver model. A temperature increase of no more than 0.11 K was observed on the surface of the investigated proximal femoral nail. The in vitro testing of orthopedic prostheses (knee and hip) revealed a slight heating effect of 0.45 K. The dependence of heating on the applied excitation frequency was measured. Overall, the tested repertoire of implants did not heat by a clinically-relevant amount in a human-sized MPI-scanner under realistic conditions, indicating their safe usage in future clinical applications.

## Abstract (web summary)

Magnetic Particle Imaging enables fast 3D imaging without radiation, ideal for cardiovascular and interventional use. Franz Wegner and colleagues tested implant heating in a perfused human cadaver and found no relevant temperature rise.

## Introduction

Magnetic Particle Imaging (MPI) is an emerging medical tomographic modality. The main principle is the visualization of the spatial distribution of superparamagnetic iron-oxide nanoparticles (SPIONs) using static and time-varying magnetic fields^1^. MPI is a background-free tracer-based imaging method offering a high temporal resolution^2–4^ and is proven to be very sensitive^5^. After its introduction as a new imaging concept, multiple preclinical studies elaborated a variety of potential clinical applications^2,6–12^. Especially, cardiovascular and periinterventional imaging revealed to be very promising^13–16^. The real-time capability of the method^17,18^ as well as the absence of ionizing radiation are very advantageous for addressing the drawbacks of widely established X-ray-based fluoroscopy techniques. In the last years, proof-of-concept studies illustrated the potential of MPI to guide the clinically most often performed endovascular interventions: balloon angioplasty and stent implantation^6,14,19,20^. Furthermore, MPI offers quantitative imaging characteristics. As the signal intensity and the tracer concentration are proportional^21^, the degree of vascular stenoses or stent lumina can be determined with high accuracy^15,22,23^. In the latter, it is well established that the majority of metallic stents do not cause image artefacts in MPI^24,25^. As clinical techniques computed tomography and magnetic resonance imaging are strictly limited regarding the lumen-quantification of stents by material induced artefacts, MPI can overcome this hurdle. The vision of radiation-free guidance of endovascular interventions became tangible with the availability of first human scale MPI-scanners and clinically approved tracer material^26–30^. A dedicated MPI-system can acquire scans of human legs in real-time and thus is the basis for MPI guided interventions (iMPI)^27^.

Extensive safety studies are crucial prior to clinical introduction of this procedure. In recent years, preclinical studies have examined the heating behavior of guidewires and stents in worst-case scenarios in a commercial small animal MPI-scanner^31–33^. In guidewires, antenna-effects can cause drastic heating. Previous studies with endovascular stents showed heating effects which were influenced by the stent design and the stent diameter. In particular, aortic stents exhibited significant heating during 430 s scans within a preclinical MPI-scanner (MPI 25/20FF, Bruker, Ettlingen, Germany, operating at excitation frequencies around 25 kHz), rendering them incompatible with human MPI examinations^34^. Regarding the upcoming clinical application of MPI for the guidance of vascular interventions, evaluations of metallic medical implants under realistic conditions are critical for ensuring patient safety. Especially, issues regarding tissue heating (*specific absorption rate*, SAR) and peripheral nerve stimulation (PNS) are of high relevance^21,35^.

In this study, we investigated potential heating of endovascular devices and orthopedic implants within a dedicated human-sized MPI-scanner operating at a main excitation frequency of 2.48 kHz^27^. To ensure the highest level of transferability, the measurements were conducted in an extracorporeally-perfused human cadaver model. Furthermore, in vitro experiments were performed in an additional setup to study the influence of the used excitation frequency on heating.

The temperature of commonly used endovascular devices (stents, coils, vascular plug) and orthopedic implants (hip and knee prostheses, proximal femoral nail (PFN)) was assessed using fiberoptic and resistance thermometers.

## Methods

### Cadaver setup

**Fig. 1:**
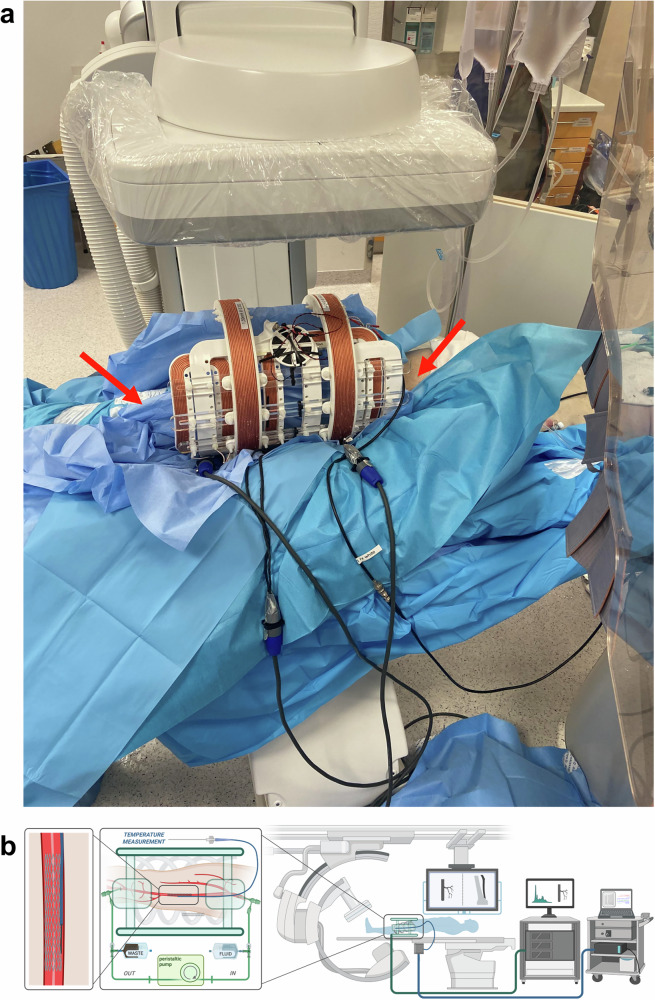
Overview of the experimental setup. **a** Cadaver leg (red arrows) centrally in the MPI-scanner. The scanner and the leg are placed between the C-arm of an angio-suite. The blue blankets around the leg protect the electronic of the MPI-scanner from moisture. **b** Schematic overview of the whole experimental setup. *Created in BioRender. Gruschwitz, P. (2025)* https://BioRender.com/f55o607.

**Fig. 2:**
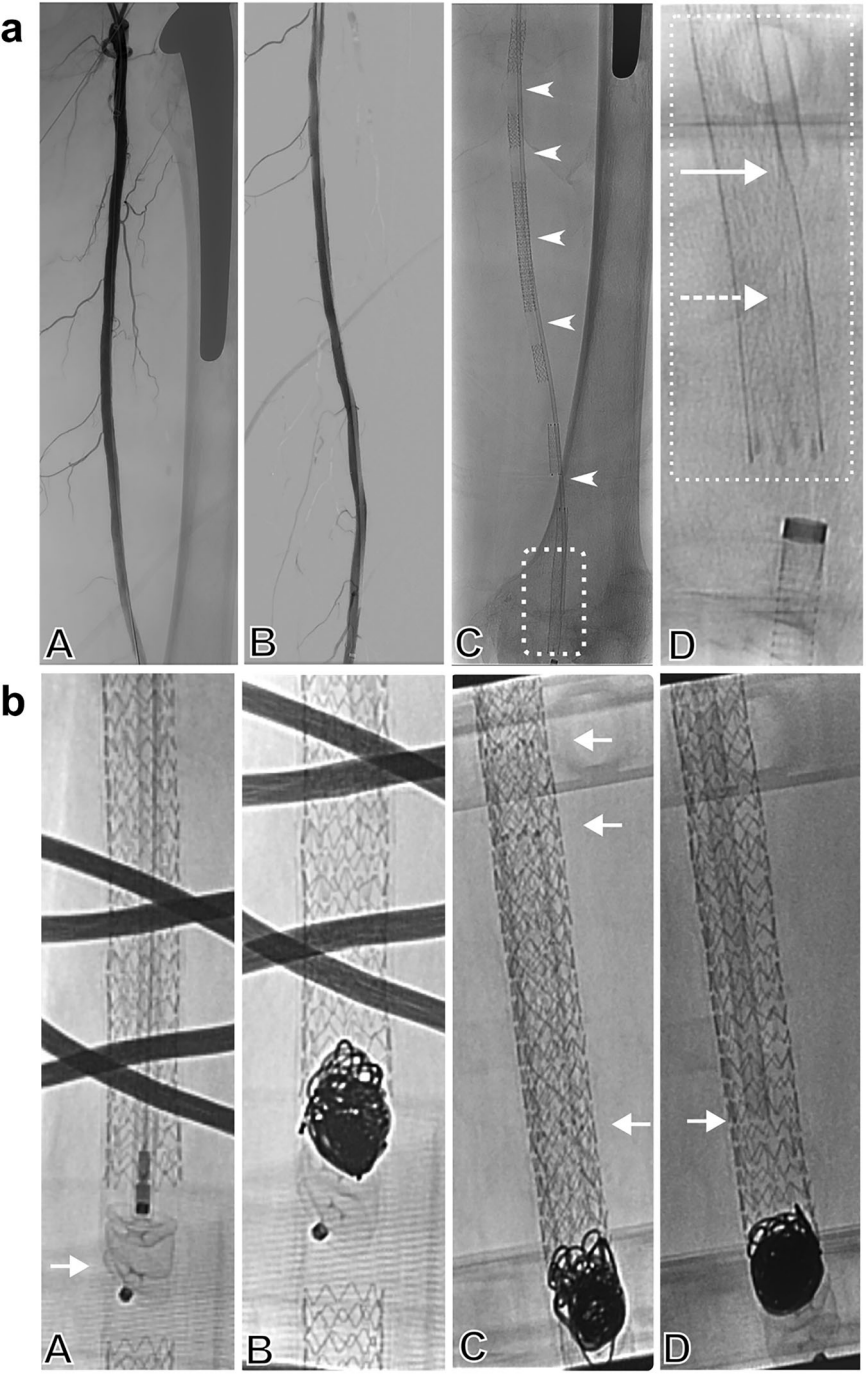
Endovascular devices in the cadaver’s superficial femoral artery. **a** A: Angiogram of the thigh after surgical preparation and setup of the perfusion circuit. Notice the patent arterial branches and the opacifying endoprosthesis in the proximal femur. B: Digital subtraction angiography after stent placement. Notice the guiding catheter in place between stents and vessel wall as protective wrapper for the temperature probe. C: Under continuous perfusion with the non-contrasted perfusion fluid, the position of the catheter and hence the temperature probe is discernible (arrowheads), whereas the position of the tip (dashed rectangle) is magnified in D: Magnification from C, whereas the catheter tip is discernible proximally (arrow) and the non-opacifying temperature probe tip protrudes 10 mm distally between the stent and vessel wall (dashed arrow). A schematic drawing of the temperature probe positioning is part of Fig. 1b. **b** A: Fluoroscopy while placing the Amplatzer Plug (arrow). Notice the overlying opacifying wires from the MPI-scanner. B: Coilbundle inside the stent with the Amplatzer Plug as anchor. C: Placement of several stents nested into each other with very dense mesh of struts. Notice the radiopaque tantalum markers indicating the stent ends (arrows). D: Temperature measurement inside the metallic stent. The end of the catheter tip (arrow) is advanced into the stent with the temperature probe tip protruding 10 mm further distally until contact with the stent is made.

The temperature measurements were partially performed in a human cadaver model. The cadaver was provided by the Institute of Anatomy, University of Würzburg. The donor of the body consented to the use of her remains for study and research purposes while she was alive. A fresh-frozen cadaver was prepared according to a published study protocol^36^. In short, after thawing, the left common femoral artery (CFA) and the popliteal artery (PA) were exposed by a board-certified vascular surgeon. Then, two 7 F vessel sheaths were inserted (antegrade CFA and retrograde PA, Cook Medical, Bloomington, Indiana, U.S.A.) directly into the vessels and fixated. The sideports of the sheaths were then connected to a peristaltic pump and a perfusion fluid reservoir containing a mixture of glucose and Ringer’s solution warmed to body temperature (37 °C) to establish a flow circuit. Due to the non-standardizable nature of the cadaver model in terms of controllable inflow, outflow, losses through side-branches, vessel wall tension and permeability, a defined flow could not be used, but the flow-rate was chosen based on visually realistic appearance on DSA as determined by two board-approved interventional radiologists. The temperature of the fluid was not actively controlled during the experimental course. A third sheath (10 F) was inserted in parallel to the perfusion sheath in the left CFA for the insertion of instruments. To guide the device implantation and the temperature measurements, the cadaver was placed in an angio-suite (Azurion 7, Philips, Eindhoven, Netherlands) with the prepared leg inside a human-sized MPI-scanner^27^ (Fig. 1). The functionality of the cadaver model was confirmed by digital subtraction angiography with the application of an iodine contrast agent bolus (Fig. 2a). The functionality of the temperature measurement setup was proven by injecting a cold (15 °C) and a warm (50 °C) water bolus (Supplementary Fig. 1). The temperature measurements were performed under two different conditions: first, under continuous flow to simulate cooling in the blood stream and second, no flow, as a worst-case scenario to detect even small temperature increases.

### Temperature measurement setup and tested devices in the cadaver model

The details of the tested devices are given in Table 1.

**Table 1:** Overview of the endovascular implants tested in the cadaver model

Device	Manufacturer/Model	Material	Size (diameter x length)
Stent 1	Abbott/Absolute Pro	Nitinol	5 mm×60 mm
Stent 2	Boston Scientific/Innova	Nitinol	6 mm×20 mm
Stent 3	Bentley/BeSmooth	Stainless steel	6 mm×18 mm
Stent 4	Boston Scientific/Express LD Vascular	Nitinol	7 mm×57 mm
Stent 5	Abbott/RX Herculink Elite	Cobalt chromium	7 mm×15 mm
Stent 6	Biotronik/Dynamic	Cobalt chromium	7 mm×25 mm
Stent 7	Abbott/Supera	Nitinol	6.5 mm×60 mm
Stent 8	Biotronik/Astron Pulsar	Nitinol	7 mm×30 mm
Stent 9	Biotronik/Astron Pulsar	Nitinol	6 mm×80 mm
Stent 10	Biotronik/Astron Pulsar	Nitinol	5 mm×40 mm
Vascular Plug	Abbott/Amplatzer Vascular Plug II	Nitinol	6 mm×6 mm
Coil 1	Boston Scientific/VortX Diamond 18 Pushable Fibered Platinum Coil	Platinum	5 mm×5.5 mm
Coil 2	Boston Scientific/Interlock Fibered IDC Occlusion System	Platinum	5 mm×80 mm

For investigating the temperature behavior of stents, first, a straight shaped catheter (5 F Radiofocus Glidecath, Terumo, Japan) was inserted in the cadaver’s superficial femoral artery (SFA) (Fig. 2a). Second, six different metallic stents were implanted one after another analogous to a stent implantation procedure in the clinical routine under X-ray guidance. After the stent implantations, the previously inserted catheter was located between the vessel wall and the stent struts. This allowed a fiberoptic temperature probe (PRB-G40-O5M-STM-MRI, Osensa, Coquitlam, Canada, measurement accuracy 0.05 K) to be directly placed at each stent by inserting it into the catheter and retracting the catheter sequentially (Fig. 1b). The temperature probe was positioned in a way that it protruded 10 mm from the catheter tip at each location. The radial force of the stents in combination with the wall tension of the vessels ensured direct contact of the temperature probe tip with the stent struts upon retraction of the catheter. The temperature probe was not moved during the measurement periods. The temperature changes were recorded with a fiberoptic thermometer (FTX-300-LUX + , Osensa, Coquitlam, Canada, measurement accuracy 0.05 K) during the MPI-scans.

After the measurements of the single stents (Stents 1−6), a vascular plug made from nitinol was inserted between two of the implanted stents (Tab. 1, Fig. 2b). The temperature probe was fixed between the vessel wall and the plug. Afterwards, a coil package was created by inserting two push coils (Coil 1) and three detachable coils (Coil 2) (Tab. 1, Fig. 2b). The temperature probe was placed inside the coil package. Finally, a worst-case-scenario was created by inserting four additional stents nested into each other (Stent 7-10) in Stent 3 (Fig. 2b). Here, the temperature probe was placed between the initially implanted stent and the first in-stent stent.

**Fig. 3:**
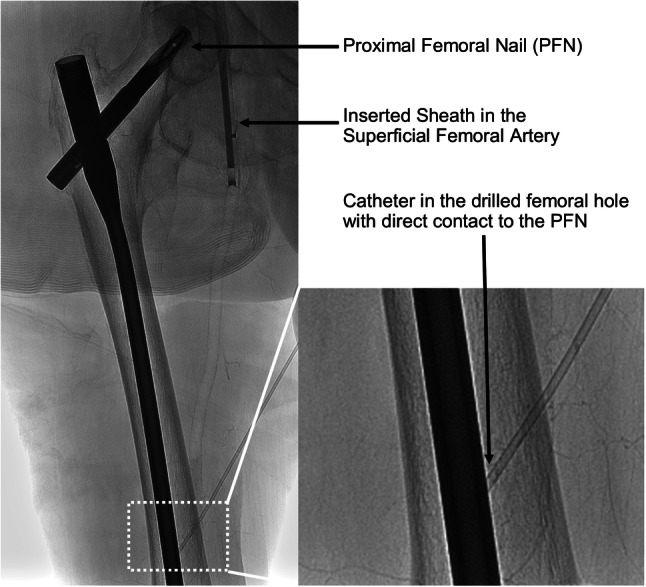
Temperature measurement setup of the proximal femoral nail (PFN). Fluoroscopy showing the PFN in the right femur with the temperature probe directly placed at the metallic implant via a radiopaque catheter (magnified image).

**Fig. 4:**
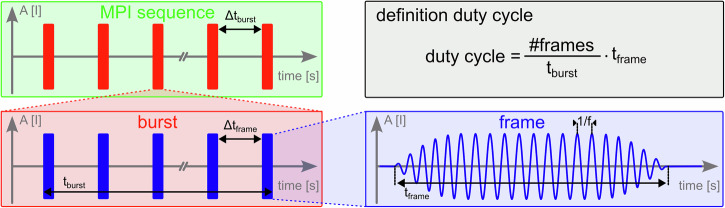
Sketch of an MPI sequence. Each sequence can consist of multiple bursts separated in time. Each burst has a defined duration t_burst_ depending on the number of frames.

**Fig. 5:**
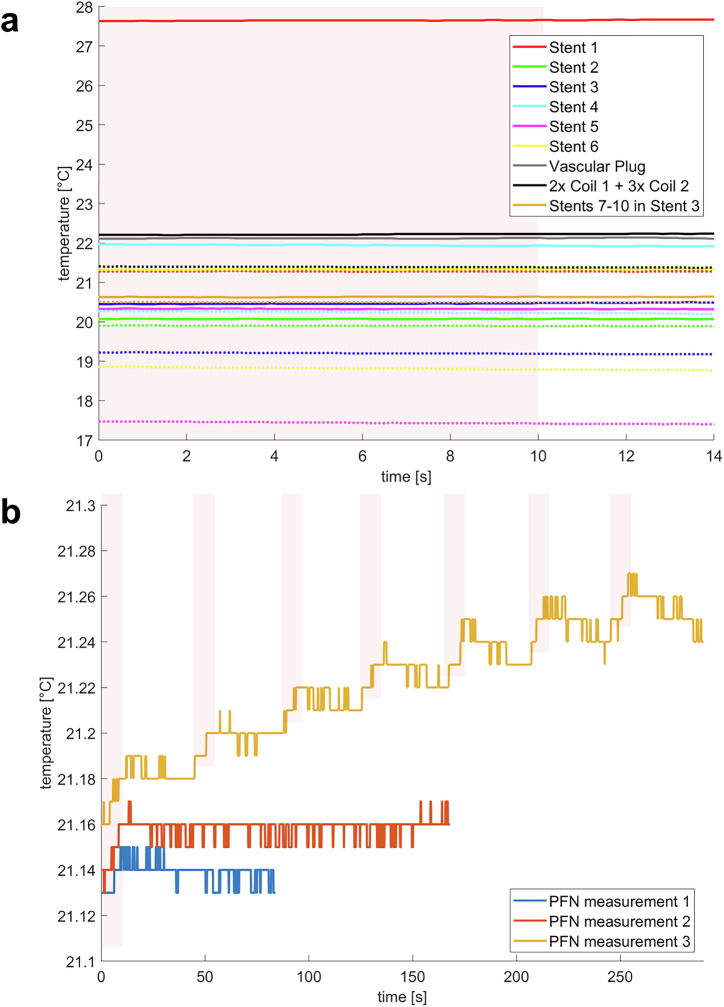
Overview of the temperature measurements in the cadaver model. **a** Temperature curves of the tested endovascular implants (dotted lines: static conditions, continuous lines: flow conditions, shaded areas: heating cycles). The varying starting temperatures are caused by a slight temperature drift of the blood equivalent fluid, which was not actively warmed during the experiment. **b** Three temperature measurements at the surface of the PFN in the cadaver model. The red area indicates the duration of the applied MPI-sequence (further details see Table 3). During PFN measurements 1 and 2 only a single sequence was applied. For PFN measurement 3 a series of seven bursts was used.

After testing the endovascular devices, the heating behavior of a PFN, which was in the cadaver’s right thigh, was studied (Tab. 2). The detailed specifications of the nail are not known due to missing individual information regarding the body donor. A hole was drilled in the right femur of the cadaver by a powered bone access system (Arrow OnControl, Teleflex, Wayne, Pennsylvania, U.S.A.) allowing direct contact of the temperature probe with the PFN (Fig. 3). The temperature probe was directly placed at the PFN via the hole and this was confirmed by fluoroscopy at multiple angles. Two measurements were performed with a single MPI-burst for initial testing of temperature increase to prevent excessive heating. Furthermore, a series of seven bursts (details see Tab. 3, Fig. 4) was applied (Fig. 5b).

**Table 2:** Overview of the orthopedic prostheses tested in an in vitro setup and the PFN tested in the cadaver model

Device	Manufacturer/Model	Material	Size
Knee prosthesis	AGC Total Knee System, Zimmer Biomet	stainless steel alloy from chromium, cobalt, molybdenum, polyethylene	femoral component: 65 mm
Hip prosthesis	Allofit-S Alloclassic, Zimmer Biomet	chromium, cobalt, molybdenum, nickel, polyethylene, and ceramic	hip socket: 48 mm
PFN	unknown due to anonymous body donor

**Table 3:** Definition of measurement protocol timings for the chosen setups

	iMPI (cadaver)	iMPI (in vitro)	Heating setup
frequency [kHz]	2.48	2.48	1.02–16.46
# bursts	1;7 (*)	10	10
Duration of burst (t_burst_) [s]	10	11	11
# frames per burst	40	10	10
Duration of frame t_frame_ [s]	0.1	0.1	0.1
∆t_bursts_ [s]	-;30 (*)	30	30
∆t_frames_ [s]	0.15	1	1
Duty cycle [%]	40	9	9

A full MPI sequence consists of multiple bursts (#bursts), which are separated in time by Δt_burst_. Each burst consists of multiple frames (#frames per burst) also separated in time by Δt_frame_ (Fig. 4). With the duration of each burst (t_burst_) and each frame (t_frame_), the duty cycle can be calculated. (*) When using a single burst (see PFN measurement 1 + 2, Fig. 5), no additional delay time is used.

### iMPI setup

For the described heating experiments, an MPI-scanner dedicated for interventional purposes was used^27^. The device is a portable, human-sized MPI-scanner specifically designed for real-time endovascular interventions^37^. It features a bore of 20 cm with an elliptical field-of-view measuring 25 cm in length, 10 cm in the minor diameter, and 20 cm in the major diameter. These dimensions are well-suited for imaging human limbs. The scanner uses a novel traveling-wave approach: two pairs of saddle coils generate a dynamic field-free line (FFL), which is then steered through the imaging volume by an additional pair of solenoid coils. The dedicated design provides an X-ray window, which allows hybrid applications alongside conventional X-ray angiography.

In terms of imaging, the scanner employs a fast 2D projection image. The FFL is moved along a sinusoidal trajectory across the field-of-view (slice-scanning mode, SSM)^4^ and data is acquired in very short time, enabling real-time visualization up to 8 frames per second^17,18^. The excitation frequencies of the SSM protocol were *f*_1_ = 60 Hz and *f*_2_ = 2.480 kHz, with a frame length of 100 ms. This rapid protocol ensures good sensitivity to the superparamagnetic iron-oxide nanoparticle tracers, providing clear, background-free images.

The protocol for the heating experiments comprises 40 images (pulses) over 10 s (4 images per second) which is comparable to the standard framerate during standard fluoroscopy (see Tab. 3) Each implant was centrally positioned within the scanner using X-ray fluoroscopy. The in vitro experiments have been performed with the same scanner.

### Measurement protocol

For the different experiments, the measurement protocol has to be defined. In Tab. 3, the frequencies, the number of frames per burst, and the bursts per MPI sequence as well as the timings are given for the different setups. Figure 4 shows the definition figuratively.

### In vitro testing of orthopedic implants (in vitro iMPI)

To gain more insights regarding the potential heating of orthopedic implants during MPI-guided peripheral interventions, two commercial implants have been investigated in an in vitro setup (Tab. 2). Therefore, each implant was placed surrounded by AgarAgar (3% mass) in a plastic container and a temperature probe (Multilayer NTC Thermistor NTCS0603E302, Vishay, Malvern, Pennsylvania, U.S.A.) was fixed directly at the implants’ surface. The experimental setup was centrally placed inside the human-sized MPI-scanner. The temperature data was recorded by a homebuilt acquisition board (PSOC 5LP–Cypress, San Jose, California, U.S.A.). To determine the measurement uncertainty of the calibrated temperature probe placed in AgarAgar, the temperature was measured over a period of 30 s without an applied magnetic field. By calculating the standard deviation of the measured temperatures, the measurement uncertainty was determined to 0.012 K.

### Investigation of the excitation frequency dependence of device heating

To test the influence of different excitation frequencies on device heating in MPI, the previously used two orthopedic implants (Tab. 2) were investigated in a home-built frequency adaptable hyperthermia-setup. This hyperthermia-setup consists of a main coil with 20 cm length and a diameter of 12 cm. For magnetic field generation, 87 windings of litz wire (400 × 0.1 mm Rupalit Classic, 1 × 52, Pack Feindrähte, Rudolf Pack GmbH & Co. KG, Gummersbach, Germany) were used resulting in an inductance of 320 µH. Using different series capacitors, the system was tuned to multiple resonant frequencies (1.02 kHz, 2.68 kHz, 4.64 kHz, 7.96 kHz, 11.72 kHz, 16.43 kHz). For all excitation frequencies, a magnetic field strength of 8.4 mT (420 µT/A) was calibrated (see Supplementary Table 1).

The setup was powered by a conventional audio amplifier (TA2400, t.amp, Thomann, Germany) and controlled by an arbitrary function generator (Arbstudio 1104, Teledyne LeCroy, U.S.A.). The data acquisition was conducted using a digital oscilloscope (HDO 8038, Teledyne LeCroy, U.S.A.). Each sample was measured with multiple bursts. Each burst consists of 10 pulses (images) each with a duration of 100 ms at intervals of approximately 10 s (total duration 11 s; duty cycle of 10%). Between the bursts a waiting time of approximately 30 s was set (see Tab. 3).

### Data analysis

In the perfusion experiments temperature measurements are reported absolutely. For the in vitro measurements temperature changes (∆T) were calculated by subtracting the temperatures before applying the MPI-sequence (T_imp1_) from the temperatures after the MPI-sequence (T_imp2_):

$$\triangle {{\rm{T}}}=({{{\rm{T}}}}_{{{\rm{imp}}}2})-({{{\rm{T}}}}_{{{\rm{imp}}}1})$$

The temperature data were processed and graphically prepared with Matlab (Matlab R2024b, MathWorks, Natick, Massachusetts, U.S.A.).

To investigate the excitation frequency dependency in hyperthermia setup, the maximum of the temperature curves for the different applied excitation frequencies (Supplementary Fig. 2) was determined and visualized in dependency of the excitation frequency (Fig. 5b).

### Reporting summary

Further information on research design is available in the Nature Portfolio Reporting Summary linked to this article.

## Results

### Device testing in cadaver model

The six commercially available stents implanted in the SFA did not show any detectable heating during the application of the MPI-sequence, neither under flow nor static conditions (Fig. 5a). The tested endovascular pushable and detachable coils as well as the vascular plug exhibited no detectable temperature changes. The created worst-case scenario of four in-stent stents in a primary implanted stent also revealed no detectable temperature changes during the applied MPI-sequences. The temperature measurements of the PFN in the cadaver’s right thigh revealed an increase of 0.04 K during a single MPI-sequence (Fig. 5b). The application of a series of seven MPI bursts showed a temperature ramp up by 0.11 K. It must be acknowledged that the observed temperature increase is in the range of the inaccuracy of the temperature measurement setup.

### In vitro testing of orthopedic implants

**Fig. 6:**
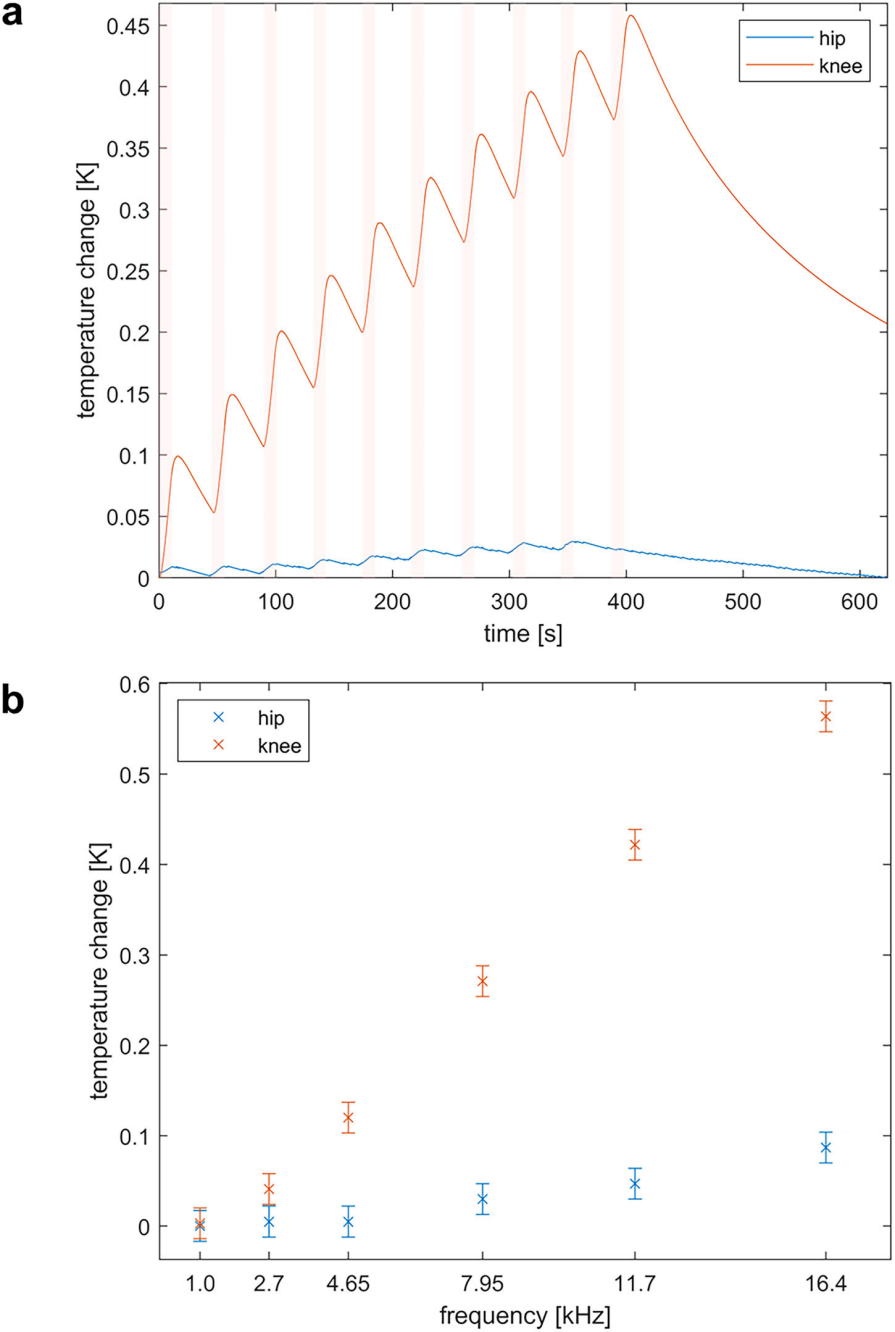
In vitro testing of orthopedic prostheses. **a** Temperature curves of the tested orthopedic implants during measurements in the MPI-scanner (red areas: heating cycles). Note: The heating cycle for the hip prosthesis was only nine bursts due to technical issues. **b** Frequency dependency of the heating investigated in a separate heating setup. The error bars result from the standard deviation of the sensor (calculated from 2734 data points) over a constant measurement period. Further sequence details can be found in Table 3.

The in vitro heat-test of orthopedic prostheses in the MPI-scanner revealed a temperature increase of 0.03 K for the hip prosthesis after a total experiment length of ~400 s (see Tab. 3). The knee prosthesis showed an increase of 0.45 K after 400 s MPI-scan (Fig. 6a).

### Investigation of excitation frequency dependency in hyperthermia setup

The excitation frequency dependency study confirmed that the temperature of the orthopedic implants increased with the excitation frequency^38^. As expected, the highest increase of 0.56 K was caused by applying an excitation frequency of 16.4 kHz for a duration of 11 s (Fig. 6b, Supplementary Fig. 2).

## Discussion

In this work, we investigated the heating behavior of a broad variety of commercially available medical implants under realistic conditions in a human-sized MPI-scanner intended for endovascular procedures of the lower extremities and observed no clinically relevant temperature changes.

The heating of metallic objects in oscillating magnetic fields, such as those used in MPI-scanners, appears to be caused by two mechanisms—magnetization losses of ferromagnetic domains and the induction of eddy currents^32,39,40^. The latter effect seems to dominate in MPI^34^, however, it is difficult to make a generalized statement here, since the effects are strongly dependent on the scanner design and the imaging parameters. The results of this work contrast with existing in vitro studies^32–34^. Previously, over 30 commercial metallic stents were tested regarding their heating characteristics in a commercial preclinical MPI-scanner. Some of the stents showed relevant heating by more than 50 K with the diameter as major influencing parameter. Therefore, particularly for stents with larger diameters than those tested in this study, more prominent heating effects would be expected. By comparing this work to the previous studies, three major differences must be acknowledged: First, the magnetic fields used in our experiments were applied in the form of bursts and had a lower excitation frequency of 2.48 kHz^27^ versus the excitation frequencies of ~25 kHz used in the experiments performed in the pre-clinical MPI-scanner, where significant heating was observed. As the resulting heating of conductive objects shows a strong dependency on the applied excitation frequency of oscillating magnetic fields^41^ the higher temperature changes of stents in the previous studies might be explained. This effect was also confirmed in the experiments with excitation frequency variation in this work. Second, the magnetic field amplitude was higher in the used interventional MPI-scanner of this work (up to 35 mT)^27^. In combination with the lower frequency, the higher field magnitudes could not replicate or exceed the heating effects observed in the previous studies since the SAR is proportional to f²*B²^35^. Third, the setup of this paper included a perfused human cadaver model^36^, while the previous studies used a worst-case-scenario in vitro setup with the tested stents surrounded by air. Thus, the perfusion fluid may cause sufficient cooling of the tested devices. As there was no difference between static and continuous flow, we assume the effect of lower excitation frequencies is more pronounced than the potential cooling of a surrounding liquid. In addition, the surrounding tissue, which consists mainly of water, has a significantly higher thermal capacity compared to the stents. However, even if heating might occur under differing circumstances using other scanner designs, blood flow would provide substantial additional cooling. A previous study described a delta of 57% between flow and no-flow for a stented blood vessel in an MRI study^42^. Furthermore, the cooling effect of the surrounding vessels must be acknowledged and is not represented in our cadaver model. Nevertheless, we observed detectable temperature increases in the tested PFN which has a higher volume of solid metal compared to the tested interventional devices. To this end, heating of metallic implants is considered safe, if it does not exceed 2 K or 39 °C tissue temperature, while some authors even claim short-lived temperature rise of up to 43 °C as upper safety limits^43,44^.

The scanner which was used in this work has been built for endovascular interventions of human lower extremities^27^. Previous work illustrated the potential to perform MPI based angiographies and established procedures like balloon angioplasties and stenting^14,19,27^. Hence, the results of this work are an essential part on the way to human application of MPI in general and especially regarding the presented scanner concept. As a variety of endovascular and orthopedic implants, which are potentially localized in the anticipated scanning region during a peripheral endovascular procedure, showed no relevant heating, a major safety concern was addressed in this work. Furthermore, it was recently proven that the clinically tested MRI contrast agent *Resotran®* has an MPI performance which is comparable to the established *Resovist®*^28^, which is not available anymore in most countries. This signifies that another milestone toward the clinical implementation of MPI has been achieved. Regarding the heating of metallic implants, we do not expect any thermal influence by circulating SPIONs during MPI measurements in the frequency range which was used in our study^45^.

Due to the given variety of (human-sized) MPI-scanners^26,27,29,30^, and their specific imaging parameters as well as field geometries, the results of our study must be carefully interpreted when transferring them to other scanner concepts. With the perspective of human application, every device should be tested in each scanner type before human usage to prevent adverse events. Therefore, the presented measurement setup in this work might be a reliable basis for the acquisition of results which are transferable to the in vivo situation. Nevertheless, we do not expect drastic heating effects in human-size scanners, due to neurostimulation limitations of the anticipated excitation frequencies and amplitudes^46^.

This study has some limitations which need to be discussed. The restricted number of assessed implants is limiting the generalizability of our results. Due to the wide variety of medical implants, this study selected a representative range of commonly used implants to reflect the typical spectrum. However, when MPI finds wider adoption, implant safety information would require extensive testing and certification from vendors, as is already done for MRI compatibility of most metallic implants. In this regard, it must be acknowledged that detailed material information on medical products is sometimes missing, which may particularly limit the mechanistic interpretation of measurements. Furthermore, the duration of the applied burst was technically limited to 10 seconds. Possibly, longer scan durations could cause detectable heating effects. However, the chosen time frame adequately reflects the duration typically required for clinical angiography procedures^47^. Active temperature control of the cadaver or the perfusion fluid was not done, which introduces an additional measurement error and diminishes generalizability of the results. Despite the presented cadaver model having a morphologically high transfer potential to the in vivo situation, a variety of physiological effects with influence on heat distribution, e.g. vasomodulation^48^, are not represented in the chosen setup. Regarding the clinical usage of the presented setup, the usability for medical staff and doctors as well as the stable performance of the system in the hospital environment must be addressed in further iterations of the so far experimental setup.

In summary, this safety study is an important step towards human application of MPI as heating of the tested medical implants in a human-sized MPI-scanner under realistic conditions remains an order of magnitude below established safety limits.

### Ethics declarations

Ethical approval was given by the Ethic Committee of the University of Wuerzburg (protocol number: 2022041301).

## Supplementary information

Supplementary material

Reporting Summary

## Data Availability

The datasets used and/or analyzed during the current study are available from the corresponding author on reasonable request.
