# Supplementary material for: Towards clinical magnetic particle imaging: safety measurements of medical implants in a human cadaver model
Source: Commun Eng. 2025 Dec 1;4:210. doi: 10.1038/s44172-025-00561-9 (PMC12673122; doi:10.1038/s44172-025-00561-9)
Supplement: Supplementary file 1 — Supplementary material [file 44172_2025_561_MOESM1_ESM.pdf]

Supplementary figures

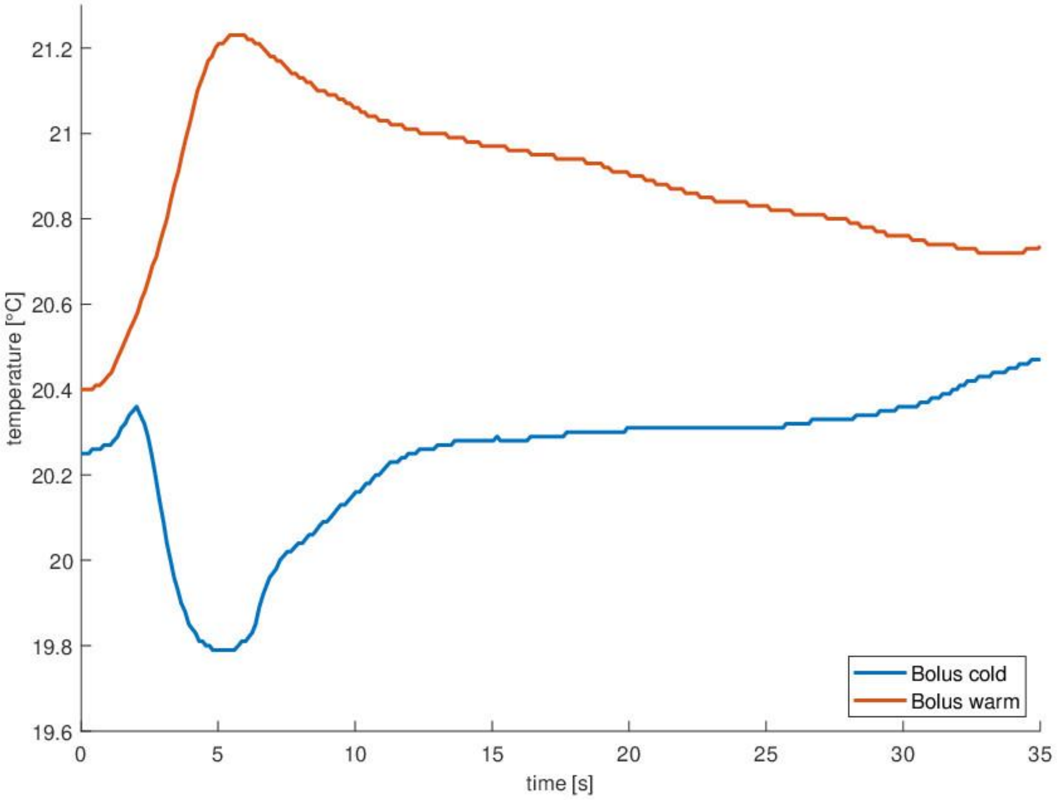

Suppl. Fig. 1: Temperature curves of a 10 ml bolus of warm (50°C) and cold water (15°C) injected into the femoral sheath of the cadaver.

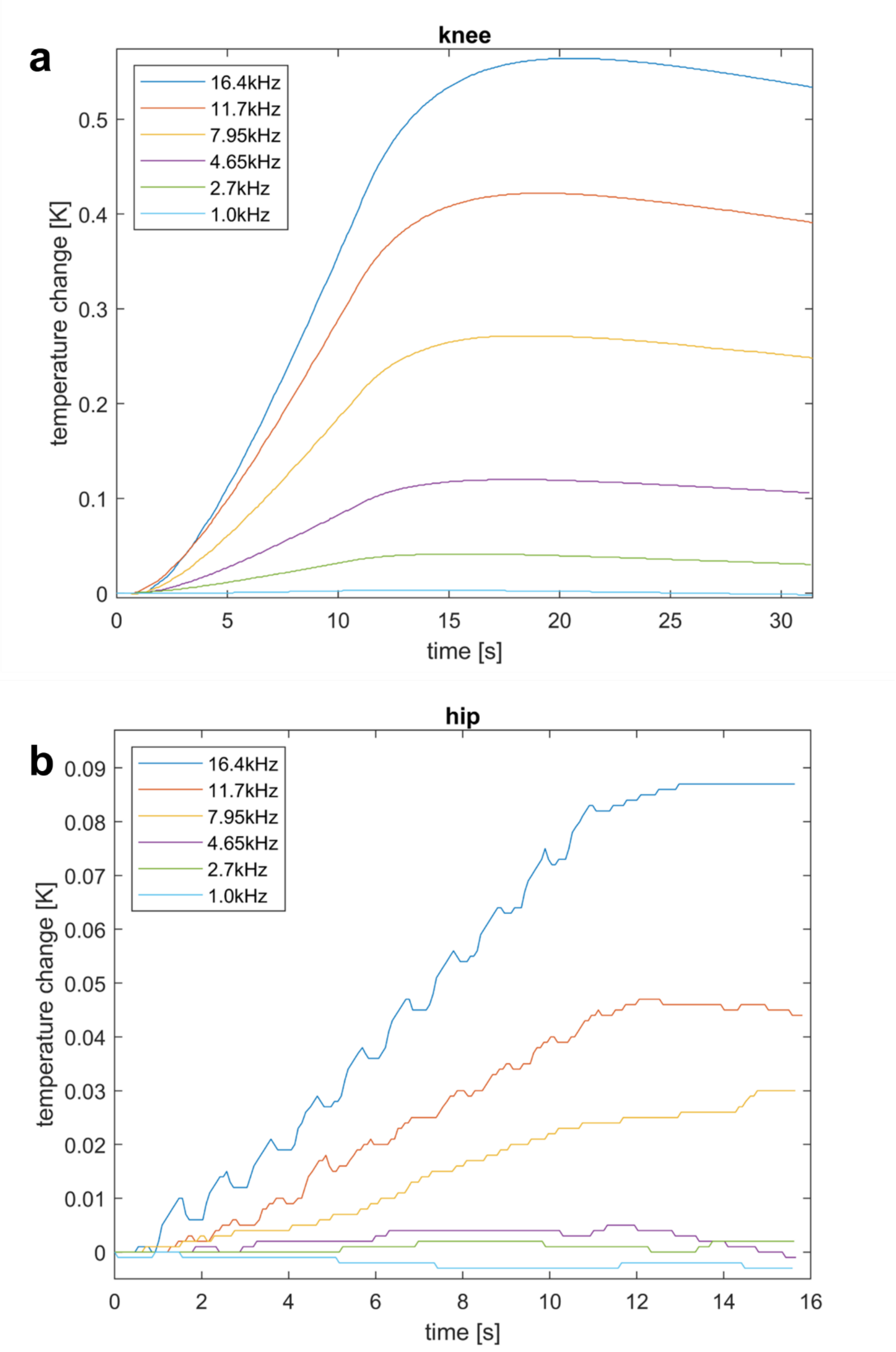

Suppl. Fig. 2: Temperature curves the tested knee and hip prostheses in the frequency varying setup. The prostheses were investigated for 11 seconds of field application with frequencies between 1 kHz and 16.4 kHz. During the measurements of the hip prosthesis sawtooth-like increase of the temperature curve was observed. This effect is assumed to be caused by an electromagnetic interaction between the applied magnetic field and the temperature sensor. The spatial orientation seems to enhance this phenomenon, as it was not observed during the measurements of the knee prosthesis.

Supplementary table

Suppl. Tab. 1: Different capacitors and resulting resonant frequencies used in the hyperthermia-setup.

Capacitance	Resonant frequency
230 nF	16.43 kHz
451 nF	11.72 kHz
981 nF	7.96 kHz
2,890 nF	4.64 kHz
8,720 nF	2.68 kHz
60,000 nF	1.02 kHz
